# Leeches Again

**Published:** 1863-01

**Authors:** 


### Editorial.

### LEECHES AGAIN.

ONE of our pet leeches died a week ago. We gave him the treatment before described as long as he lived. It is better, practically, than that of T. After use, his lie idle and stupid till digestion is nearly completed, which requires one, two, or three weeks. Ours are ready for use, (and have often been used,) in from twelve to thirty hours. In salting them, to make them disgorge, they should be put in a vessel containing nearly water enough to cover them. The water protects their sides from the action of the salt. The fine table salt should be used; and it should be first applied on the posterior portion of the back, and gradually sprinkled along the back till it almost, (but not quite,) reaches the head. Let the leech crawl about in the water till it disgorges, (from two to five minutes,) then wash it with clean rain water, and return it to its home. Give it fresh rain water two or three times a week.

Our late *pet* lived with us, under this treatment, thirteen months. His mate is still alive and well, and is a first class sucker. W.

